# An AI approach to lunar phase detection: enhancing the identification of the new crescent with astronomical data integration

**DOI:** 10.3389/frai.2026.1727824

**Published:** 2026-02-13

**Authors:** Murad Al-Rajab, Samia Loucif, Raed Abu Zitar, Mubarak Gwaza Abdu-Aguye

**Affiliations:** 1 College of Engineering, Abu Dhabi University, Abu Dhabi, United Arab Emirates; 2 College of Technological Innovation, Zayed University, Abu Dhabi, United Arab Emirates; 3 College of Engineering and Computing, Liwa University, Abu Dhabi, United Arab Emirates; 4 Mohamed bin Zayed University of Artificial Intelligence, Abu Dhabi, United Arab Emirates

**Keywords:** astronomical images, deep learning, lunar crescent detection, lunar cycle recognition, machine learning, new crescent identification, occlusion in lunar detection

## Abstract

**Introduction:**

The observation of the lunar crescent is significant in astronomy, cultural traditions, and religious lunar calendar determinations. However, earth-based imaging that captures all lunar phases, particularly the new crescent across multiple months, remains limited. This study explores the feasibility of using artificial intelligence (AI) techniques to detect and analyze the birth of the new lunar crescent using space-borne imagery from NASA’s Lunar Reconnaissance Orbiter (LRO), spanning over 13 years.

**Methods:**

This study evaluates both deep learning and traditional machine learning approaches for new crescent detection. Convolutional Neural Networks (CNN), Random Forests (RF), and Support Vector Machines (SVM) were applied to orbital lunar images. A custom image preprocessing pipeline was implemented, including grayscale conversion, contrast-limited adaptive histogram equalization, and noise reduction. The CNN architecture was further enhanced by integrating lunar imagery with moon age data. Experiments were conducted using a temporally split dataset to simulate real-world conditions. Model robustness was also evaluated using synthetically generated noise and occlusion.

**Results:**

The experimental results demonstrated high performance across all evaluated models, achieving precision, recall, F-score, and overall accuracy of approximately 98%. Among the tested approaches, RF and CNN models produced the best overall performance, outperforming SVM. The CNN model showed strong robustness under degraded image conditions, maintaining high accuracy when subjected to Gaussian noise and image occlusions of up to 50%.

**Discussion:**

The findings indicate that AI-based techniques, particularly CNN and RF models, are effective for detecting the new lunar crescent from orbital imagery. The robustness of the CNN model suggests practical applicability in real-world lunar observation scenarios. This study contributes toward supporting traditional crescent identification methods and offers potential solutions for reducing calendar discrepancies across different regions.

## 1 Introduction

The appearance of the lunar crescent is so important in astronomy, cultural traditions, and religious actions. The first visible waxing crescent happens after the moon conjunction. So, the terms “new crescent” and “first visible waxing crescent” are used interchangeably throughout the paper. The Islamic lunar calendar, which regulates events like Ramadan and the Islamic New Year, depends entirely on sighting the new crescent moon to start each month. Even though this is a tedious process, it remains extremely difficult to sight the new crescent moon, and so we see continued disparity in Islamic dates across different regions of the world. This is due to challenges related to varied observational conditions. Factors such as atmospheric interference, geographical differences, and the subjective nature of human sighting contribute to these challenges.

Historically, the new crescent moon identification has been determined by human observation along with basic astronomical principles. However, the procedure contains tendencies to alteration because the weather conditions never remain constant; the precision of instruments used sometimes leads to error while recording various activities; and the observer’s location subsequently alters such data. This often leads to various Muslim communities around the world starting Ramadan and other significant religious months on different days, causing confusion and debates. Additionally, this problem is even more vast since conventional models primarily employ visual sightings and do not always account for the full range of environmental conditions that can impact visibility.

The traditional approach to crescent moon sighting relies on astronomical calculations involving parameters such as the age of the moon when it aligns with the sun, its angle from sunset and sunrise that is above some specified height, and angular differences in position compared to earlier observations, also known as the Arc of Light. These methods have been refined over centuries, from early Babylonian astronomy (van Gent, 1998) to Muslim scholars like Al-Khawarizmi and Al-Biruni (Pingree and Bīrūnī, 2010) who established criteria for deciding new moon visibility. Despite these advancements, traditional methods remain limited in addressing complex observational challenges such as cloud cover, light pollution, and variations in the observer’s proficiency and equipment.

One of the most significant disadvantages of relying on manual observations is the subjective nature and inconsistency it introduces. Measurements can vary between observers depending on their location, the sky clarity, or the optical instruments employed. These limitations can sometimes not even be surpassed by advanced telescopes and imaging devices, as atmospheric conditions may not always allow us to see the new crescent moon. As a result, there is often a lack of uniformity and inconsistency in determining the start of key religious months in Muslim countries that still rely on the traditional moon sighting practices.

AI has recently proven to be promising to address these crescent identification challenges (Tafseer, 2020; Utama et al., 2023; Muztaba et al., 2023). In light of these constraints, data-driven approaches have been developed to improve the new crescent identification. These methods aim to complement scientific prediction models by including environmental information like weather conditions and geographical location. Recent studies have found that accounting for factors like the moon age, distance from the sun, and weather conditions can enhance predictions of new crescent moon appearance, complementing traditional methods.

This paper seeks to advance these methods by incorporating lunar and environmental information to identify the new crescent. Due to the lack of earth-based images capturing the different phases of the moon within a single month and across multiple months, including the new crescent, this research employs orbital images based on data from NASA’s LRO spanning over 13 years. Unlike previous works that relied on manual descriptions or basic astronomical calculations, the objective of this study is to investigate whether AI models can learn and identify patterns related to the birth of the new crescent moon from images. In this work, we propose a novel approach to identify patterns of new crescent moon birth by combining ML and DL algorithms, including CNN, RF, and SVM, with astronomical data. However, this approach will not replace traditional moon sighting as dictated by religious practices; rather, it serves as support for the traditional sighting of the new crescent moon. This work provides a foundational step toward automated lunar phase analysis and may support future efforts that integrate terrestrial observational constraints.

The rest of this paper is organized as follows: Section 2 covers the background and related work on new crescent moon sightings and data analysis techniques. Experimental methodology and setup including the Data collection and analysis are described in Section 3. Section 4 presents the results of our experiments and discusses the findings in the context of real-world lunar observation challenges. Finally, Section 5 concludes the paper and offers insights into future research directions for improving the accuracy and reliability of the identification of the new crescent.

## 2 Background and literature review

### 2.1 Background

The standard lunar cycle consists of eight ‘phases’, each with a distinct visual appearance. For instance, at the beginning of the lunar cycle (also called the conjunction or the ‘birth of the new moon’), no part of the moon is visible to the naked eye. Its visible/illuminated area steadily increases up until the middle of the lunar cycle when it is possible to see the entirety of the moon (commonly termed the ‘full moon’ phase). Afterward, the visible area decreases again till the moon completely disappears. Notably, the visual appearance of the pre-full moon phases (termed the waxing phases) and the post-full moon phases (termed the waning phases) are anti-symmetrical (Hogendijk, 1988; Bruin, 1977).

In practice, the beginning of a new lunar month is signaled by the direct sighting of the moon’s first visible waxing crescent phase (which is the first visible phase in the lunar cycle) by human observers. Furthermore, this process holds significant historical and cultural value, not to mention its particular significance in the Islamic religion (Odeh, 2004; Faid et al., 2024). However, the first visible waxing crescent phase has the smallest visible area on the moon’s surface. This makes it difficult to observe, especially when considering difficult atmospheric conditions such as rain or fog, as well as criteria affecting the visibility of the moon based on the geospatial position of the observer. Therefore, disputes between reporters of lunar observers are common, leading to notable schisms across the Islamic world, e.g., a lack of uniformity in the Islamic calendar across different regions, etc. To mitigate this and related issues, most lunar observation is done professionally with specialized equipment, e.g., at astronomical observatories, drastically eliminating differences in opinion.

### 2.2 Literature review

Lunar detection, prediction, and classification have been the subject of many research works during the previous years. It has great importance for many religious, cultural, and social events for people on earth. Many techniques were used for this purpose including regular calculations, ML techniques based on quantitative measurements, computer vision techniques based on the lunar images, in addition to DL methods. Numerous studies have introduced requirements and classical Islamic and astronomical sighting criteria for determining and identifying the new crescent moon. Among these are Danjon’s criterion on minimum elongation (Danjon, 1932), Yallop’s visibility model based on arc of vision and altitude (Yallop, 1997), and Odeh’s integrated criterion combining crescent width and altitude (Odeh, 2004). All these criteria have been incorporated into numerical datasets when applying ML/DL techniques for this purpose. In the following, we present a summary of a literature review of some works that tackle this issue.

Fakhar et al. (2014) work aim to detect the crescent moon from input images using a two-stage, parallel approach. Initially, the image noise is removed using Gaussian smoothing, after which the proposed parallel stages are applied to this smoothed image. The first stage involves enhancing the image contrast using adaptive local histogram equalization (CLAHE), yielding the first output. The second stage performs circle detection using the Hough transform (after Canny edge detection) to generate a candidate for the center of the detected circle (i.e., the moon). A human user then compares the two results to make the final decision. The authors provide subjective proof (via images) of the efficacy of each stage.

Furthermore, Sejzei and Jamza (2016) consider the problem of subjective crescent moon detection from images acquired via telescopes or Charge coupled devices (CCDs). They investigate multiple enhancement techniques cutting across spatial and frequency domains for improving the visibility of the new crescent moon. Then they assess the suitability of each algorithm by a subjective test involving a total of 30 people. The results they obtained indicate that power, local brightness, and adaptive histogram enhancements provided the best benefit for the stated task. The authors also create MATLAB-based software including all the algorithms considered for future use.

In addition, Ali and Khidhir (2021) tackle the problem of lunar daytime crescent image enhancement under daytime conditions. They propose the use of a Wiener filter to enhance/clarify the images. They evaluate their approach on a self-collected dataset and provide subjective results indicating its performance. While Moshayedi et al. (2022) propose an algorithm called Sunfa Ata Zuyan (SAZ) for moon phase detection from images. It consists of 7 stages: color space conversion, regions of interest (ROI) extraction, noise cancelation, edge detection and resizing, radius computation, circular coverage, and moon phase regression. Their algorithm is based on the idea that the moon phase, they consider the standard 8 moon phases, can be determined by the part of the moon image that is illuminated. They evaluate their algorithm on two test sets: one is a synthetic, fixed benchmark consisting of 27 images. The other is a set of 8 images taken by an embedded platform they designed for this purpose.

Moreover, Zulkeflee et al. (2022), propose a simple, four-stage pipeline for identifying the crescent moon region given a lunar image. The pipeline performs a color-space conversion (RGB to grayscale, not to Hue Saturation Value (HSV) followed by contrast enhancement CLAHE. After that, the image undergoes noise removal using a bilateral filter, after which the image is cropped to center the crescent. After this preprocessing, the maximally stable extremal regions (MSER) technique is used to formally detect the ROI corresponding to the crescent moon. The detected ROI is further post-processed by using a geometric threshold based on elliptical major axis length for better discrimination. The proposed system shows high scores (90%) across three similarity indices ranking the overlap between the predicted and actual crescent regions.

On the other hand, Muztaba et al. (2024) focus specifically on automatic crescent detection from images. They consider the collected data (from the listed sources) as positive samples, and collected images of the landscape and related objects as negative examples. Similar to previous work, they propose a 2-stage pipeline: preprocessing and inference. Preprocessing involves grayscale conversion, CLAHE, and the Zero to Threshold method. Next, Haar features are extracted and used to train an AdaBoost classifier. The authors offer a qualitative evaluation of their system showing that it is able to detect the moon even from cellphone screens.

Additionally, Jawahir et al. (2022) aims to enhance the visual clarity of lunar images by proposing the application of known image enhancement techniques. They propose a conversion into the LAB color space, followed by CLAHE in that space, and then convert the image back into the RGB space. Afterward, the image is cropped and contrast-enhanced, then finally denoised using a bilateral filter. They evaluate their algorithm on a self-curated dataset and provide qualitative and quantitative results using Peak Signal-to-Noise Ratio (PSNR)/ Mean Squared Error (MSE) showing the efficacy of their method.

Furthermore, Utama et al. (2023) propose detecting the young lunar crescent from video data using pure Computer vision (CV) techniques. Their approach is designed to work on a frame-by-frame level and consists of several stages as follows; given an input frame, they preprocess it by converting it to grayscale, blurring it then converting it to binary and filtering it using opening operations. Next, object filtering is carried out by removing objects whose convex hull area is less than some threshold, leaving just the arc of the crescent. Finally, a circular Hough transform is used to detect the presence of a circle corresponding to the moon in each frame. The authors report an average accuracy of 79.19%.

In addition, Muztaba et al. (2023) employ a segmentation model called Mask- Region-based Convolutional Neural Network (RCNN) to detect and localize the moon in the lunar images. They incorporate this model, which uses a ResNet101 backbone, into their proposed framework, a desktop program called “Realtime fasting crescent detector.” The authors notated the datasets manually and considered different lunar phases as distinct objects with the waxing crescent being the key target. They experiment with three data distribution settings and different training regimes (i.e., full vs. head finetuning and different hyperparameter settings) to obtain optimal parameters. In validation, the authors also examine the behavior of the RCNN on different input images to ensure that it can accurately identify the crescent. The authors report good results (via the average precision and mean average precision metrics) ranging from 0.88 to 0.99. Furthermore, they claim tentatively that the system can function even in daytime and cloud-occluded settings. Therefore, they carried out a live test on the start of Ramadan 1,444 AH to evaluate their system, backed by tests confirming the identification of the new crescent on that day. The proposed system was able to detect the new crescent on that day, confirming its efficacy.

Finally, Al-Rajab et al. (2023) use ML algorithms to determine the start of Ramadan month (an Islamic month) relying on the visibility of the new crescent moon. The results based on the RF and SVM models showed very accurate prediction and evaluation performance as compared to the other ML models considered in that study. Finally, Loucif et al. (2024) use classical techniques of ML and DL based on numerical data to predict the crescent moon birth. They divided the geographical areas into different segments and used ML hybridization with metaheuristic techniques to improve the results. The authors applied cross-validation to evaluate the learning process and implemented different testing comparisons. It turned out that decision tree-based methods showed the best results.

In summary, this study sets out to predict the appearance of the new crescent moon using image-based techniques rather than relying on numerical data, which, to the best of our knowledge, is a novel approach in this field. While previous research has focused deeply on the birth of the crescent moon detection, prediction, and classification, employing methods ranging from traditional calculations to ML and DL, these approaches have primarily focused on numerical data or subjective manual techniques. Such methods are pivotal for important religious, cultural, and social occasions globally. In contrast, our study shifts toward an image-centered methodology, offering a new avenue for automating and improving the prediction of the new crescent moon.

## 3 Experimental methodology and setup

In this section, we describe our experimental approach and the details of our experimental setting, including evaluation datasets, metrics, and methodology.

### 3.1 Problem statement and motivation

As discussed in Section 1, we aim to identify the beginning of the lunar month given astronomical observations of the moon. Based on the preceding background, it is clear that:

- During the birth of the new moon, the moon is visually unobservable. This is the exact beginning of the lunar month.
- The moon’s visibility steadily increases from completely invisible to the full moon, after which it returns to invisibility, signaling the initiation of a new lunar cycle.

Based on these observations, we propose to identify the start of the lunar month by jointly considering the moon’s visual appearance and its corresponding age simultaneously. Our motivation for this approach lies in the fact that the visual appearances per phase are not unique (due to the symmetry described previously) and are affected by lunar liberation, making visual appearances unreliable as a sole source of information. By augmenting with auxiliary information (such as the age of the moon), we aim to obtain a more accurate estimation of the actual start of the lunar month.

### 3.2 Data acquisition, annotation and preprocessing

Due to the difficulty in sourcing lunar images with the required corresponding annotations, we consider data obtained from NASA’s Scientific Visualization Studio2. This is a publicly- available resource hosting (among others) color lunar imagery and associated data (e.g., moon age, moon illumination percentage, etc.) collected from January 1st, 2011 till date. The data was acquired every minute from the start date using NASA’s LRO. The images are gamma-corrected, white-balanced, and range-adjusted for similarity to human vision.

**Figure 1:**
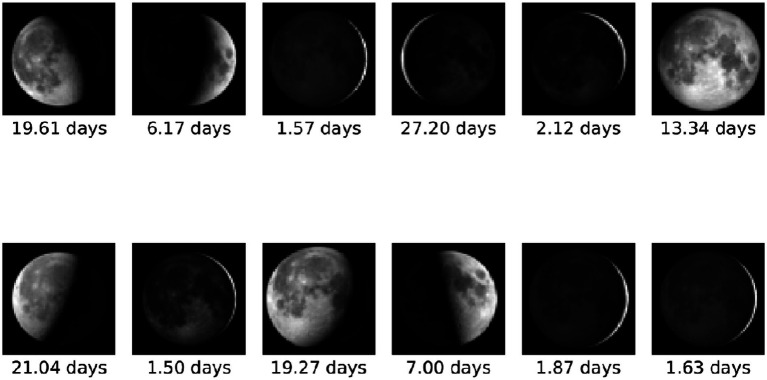
Sample of images and their corresponding moon ages (https://svs.gsfc.nasa.gov/).

We consider data over a 13-year period (from the data start date till July 31st, 2024). Due to the large data volume, we randomly sample four images per day, with each of these four images randomly taken from successive six-hour quadrants in the day (i.e., the first image is randomly selected from a time between midnight and 6 a.m., the second is randomly selected between 6 a.m. and 12 noon, etc.). Utilizing this strategy, we obtain a total of 19,844 images and their associated details. Images and their associated moon age at acquisition are considered as inputs to our proposed system (samples shown in Figure 1).

To annotate the samples as positive or negative, we utilize a threshold criterion based on the moon’s age; that is, the first visible waxing crescent phase comes when the moon is roughly a day to two and a half days old. Therefore, we label all images acquired within this age range as positive, and all other images (i.e., outside of this range) as negative.

Since we consider the start of the lunar month as the positive samples, our problem setting naturally leads to a severe imbalance in the number of data samples in favor of the negative class (i.e., other moon phases). To combat this, after splitting the data into training and testing sets (discussed subsequently), we randomly under-sample the negative samples in both sets to produce perfect balance (i.e., equal numbers of positive and negative samples). This allows the chosen downstream classifier (discussed subsequently) to learn without bias, and allows for its fair evaluation, as it must be capable of correctly classifying both positive samples (to reliably signify the start of the month) and negative samples (to avoid providing spurious signals).

It is worth noting that when using human observation data from the Earth, we consider important parameters such as elongation, altitude difference, sun–moon lag, DAZ, azimuth difference, etc. However, in this study we are using images from NASA’s LRO, so we focused only on the moon age as the main feature, which has been used in both astronomical and calendrical studies as a baseline organizing variable (feature) for early crescent analysis. Applying the above-mentioned criteria directly to LRO-based imaging would introduce assumptions that are neither measured nor required in the present approach.

We preprocess the images by converting them to grayscale and resizing them to 64×64 pixels, which maintains visual quality while reducing computational overhead. We discuss how feature extraction and classification are carried out in the following section.

### 3.3 Feature extraction and classification

In this work, we consider two families of predictive models. We begin by experimenting with traditional ML classifiers, e.g., RF (Kelleher et al., 2020) and SVM (Sarang, 2023), due to their ubiquity, low data requirements, and performance. In particular, we consider their use in conjunction with three feature extraction techniques (for the image component per input sample; the moon age component is concatenated to the computed image features): Histogram of Oriented Gradients (HOG) (Tomasi, 2015), Gray Level Co-occurrence Matrix (GLCM) properties (contrast, dissimilarity, homogeneity, ASM, correlation), and Local Binary Patterns (LBP) histograms, due to their ubiquity, low data requirements and performance. In particular, we consider their use in conjunction with three feature extraction techniques (for the image component per input sample; the moon age component is concatenated to the computed image features): HOG, Gray Level Co-occurrence Matrix (GLCM) properties (contrast, dissimilarity, homogeneity, ASM, correlation) (Ojala et al., 1994), and LBP histograms (Loshchilov and Hutter, 2017). These are selected due to their general popularity for computer vision tasks. We provide the details of the configuration of each feature extractor and classifier in Table 1.

**Table 1:** Configuration details/hyperparameters of feature extractors and traditional classifiers.

Type	Method	Parameter	Value
Features	HOG	Orientations	9
		Pixels per Cell	4
		Cells per block	2
		Scale Ration	1/3
	LBP	Radius	1
		Number of Points	8
		Method	Uniform
	GLCM	Pixel offset	1
		Angles	0°, 45°, 90°, 135°
Classifier	SVM	Kernel	RBF
		Margin Constraint ©	1.0
	RF	Trees	100
		Criterion	Gini Index

Additionally, to the best of our knowledge, this is the first study that applies ML and DL techniques to identify the birth of the new crescent moon using image-based analysis. Furthermore, no publicly labelled Earth-based datasets of observations are available for this type of study. Due to this, we resorted to using orbital images obtained from NASA’s LRO, which provide consistent, high-resolution imagery suitable for computational analysis. In this exploratory context, we applied deep CNNs (Krizhevsky et al., 2012), which have seen widespread adoption in multiple domains (Yamashita et al., 2018; Kalchbrenner et al., 2014; Acharya et al., 2017). This is due to their ability to model highly complex problems with good predictive fidelity and without the need for explicit feature engineering (unlike the traditional classifiers in our initial experiments). Due to the small training set size and the novelty of the problem domain, we designed a small CNN architecture to alleviate overfitting issue while benefiting from the representational power of DL techniques. The inclusion of both classical ML models and CNN-based approaches allows us to establish baseline performance and assess the feasibility of applying image-based learning to this previously unexplored astronomical problem.

**Figure 2:**
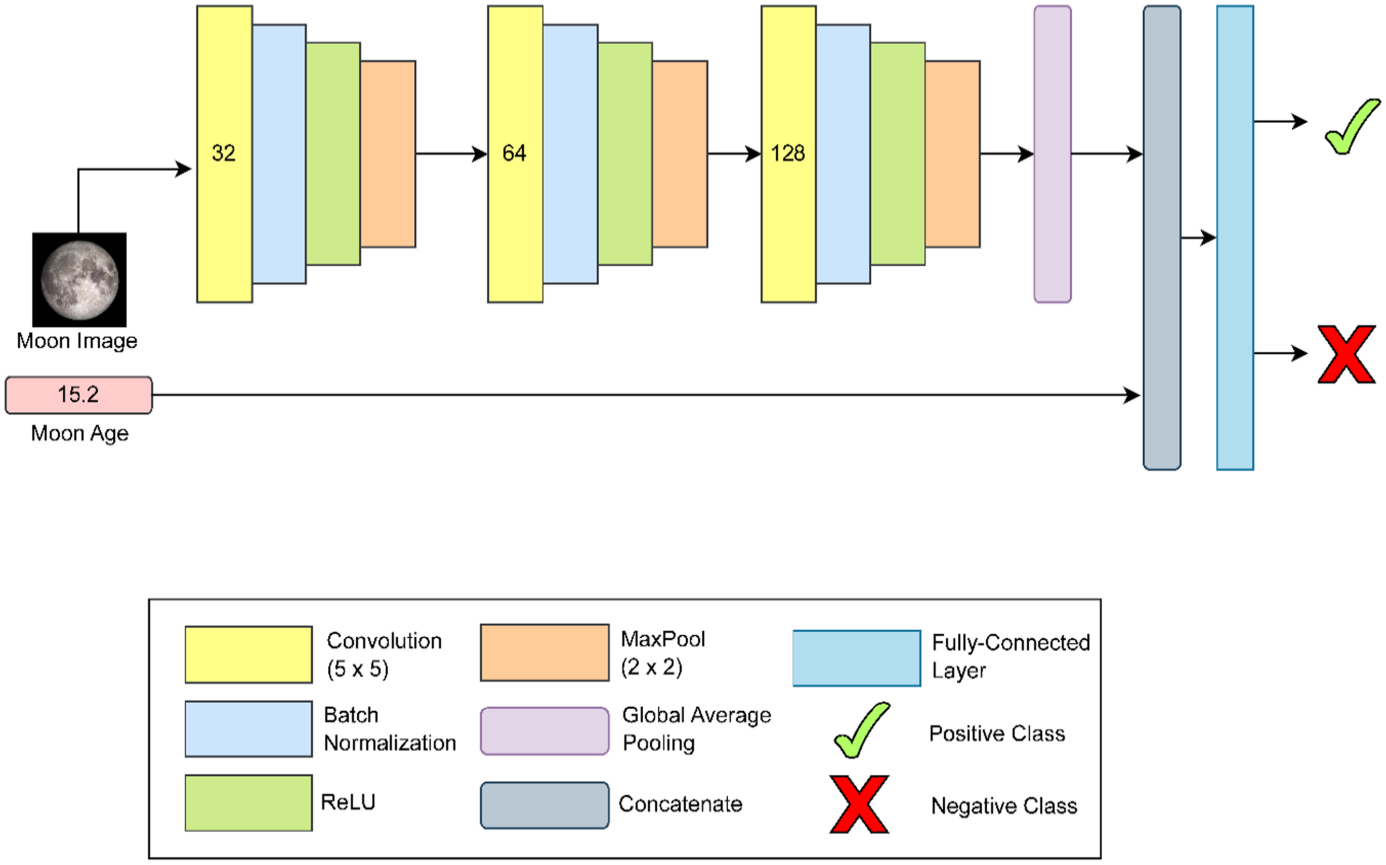
Architecture of CNN model. Numbers in convolutional blocks (yellow) indicate number of output filters.

As illustrated in Figure 2, our proposed CNN architecture receives an image of the moon and its corresponding moon age as inputs. Accordingly, the network consists of two branches dedicated to visual and scalar processing, respectively. The visual branch, which, is responsible for the image feature extraction, is composed of three successive convolutional blocks, each block is a convolution layer with a (5 × 5) kernel and has an increasing number of filters (32, 64, and 128, respectively), followed by batch normalization and a ReLU non-linearity to improve convergence and introduce activation sparsity. Furthermore, each block includes a (2 × 2) max-pooling layer to reduce spatial dimensionality and control overfitting. The resulting feature maps are aggregated into a compact image-level representation using a global average pooling operation (Zhong et al., 2020). This image-based representation is then concatenated with the moon-age feature and passed to fully connected layer for final binary classification into positive and negative classes. Based on the widely adopted CNN design convention, a stride of 1 for the convolutional layers and a stride of 2 is used for the max-pooling operations.

The intermediate representations from the visual branches are then concatenated with the output of the scalar branch, after which the combined representation is passed through a one-layer fully-connected classifier to produce a binary output (i.e., start of month: positive or not start of month: negative). Therefore, we train the model to minimize the binary cross entropy loss (BCE) in the standard way, i.e., using minibatch gradient descent. The parameters of the training process are shown in Table 2.

**Table 2:** Neural network training configuration.

Parameter	Value
Optimizer	AdamW [24]
Batch size	16
Learning rate	1e-3
Epochs	100

### 3.4 Evaluation protocol and metrics

In evaluating all our predictive models, we consider a temporally split approach. That is, the samples used in testing the model are obtained from a completely different time horizon than those used in training. This is aimed at evaluating the model in as close to real-world conditions as possible, i.e., since it will be trained on historical data, to make predictions on future data. In this work, we set the split date for the testing portion at 1st January 2021, i.e., all samples before this date constitute the training set, and all samples including and after this date constitute the testing set. After splitting and balancing the training and testing subsets, we obtain a training set size of 829 samples and a testing set size of 342 samples (approximately 70:30 train-test ratio).

Since the testing set is perfectly balanced, we consider accuracy as the primary performance metric. This is done as accuracy intuitively provides a rate at which the model correctly classifies both positive and negative samples. We also investigate per-class and macro-averaged precision, recall and F-measure as secondary evaluation metrics for additional and granular insight.

For all predictive models considered, we repeat model training and evaluation five times just as a heuristic (random) value to repeat the training and average the results. Performance metrics obtained during each run are then averaged to provide statistically valid results and measures of dispersion (specifically standard deviation).

### 3.5 Practical implications for lunar calendar determination

Our proposed AI model can assist in decision-making for lunar calendar determination. By incorporating the observer’s geographical location and the corresponding environmental conditions, the model estimates whether the visibility of the new crescent and the age of the moon are consistent with the onset of a new lunar month.

Lunar crescent observation holds deep cultural and religious significance in many societies, where calendar determination is governed by long-established traditions and institutional authority. The proposed model is not intended to replace traditional or religious sighting practices; rather, it provides a support and explainable complementary decision-making tool. This is particularly valuable in regions where conflicting lunar determinations are reported, especially when a one-day difference in the lunar calendar may lead to social and religious inconsistencies. However, final calendar determinations remain the responsibility of authorized bodies, consistent with established practice.

## 4 Analysis of training and testing results

The ML models in this study consist of two primary types: DL, represented by a CNN, and classical ML, represented by a RF and a SVM. The DL model CNN operates directly on images, performing implicit feature extraction and classifying images into either “first visible waxing crescent” (positive) or “other lunar phases including later waxing and waning” (negative). In contrast, the classical ML models, RF and SVM, require explicit feature extraction prior to classification.

The CNN, as a DL model, demands relatively large datasets to capture details across different circumstances effectively. However, this model does not require a separate preprocessing stage for feature extraction, as its convolutional layers handle this task inherently. To suit the relatively small dataset available here, a moderate-sized CNN was selected to prevent overfitting and ensure efficient processing. Hyperparameters were tuned empirically to optimize performance, adjusting factors like learning rate, batch size, and the number of epochs.

For the classical models, SVM and RF, an additional preprocessing stage was implemented to extract relevant features from the images. The SVM model identifies an optimal hyperplane that separates the classes with minimal error, while the RF model is decision tree-based method that keeps updating the trees randomly to reach best decisions. This combination of methods, each differing in philosophy and operation, provides a comprehensive framework for evaluating and comparing ML techniques.

For feature extraction, various methods were employed to maximize the effectiveness of the classical techniques. HOG statistically analyzes image gradients, LBP capture the local spatial structure of the image, and GLCM assesses texture by analyzing gray-level dependencies. These methods offer diverse perspectives on the images, enabling more robust classification performance.

Table 3 presents the comparisons between these ML models, utilizing metrics such as accuracy, precision, recall, and F-measure. The positive class for these metrics is defined as “first visible waxing crescent.” To address class imbalance, the dataset was under-sampled to create a balanced representation, which helps improve the reliability of accuracy and other performance metrics.

**Table 3:** Experimental results.

Metric	Method						
	HOG+RF	HOG+SVM	LBP+RF	LBP+SVM	GLCM+RF	GLCM+SVM	CNN
Accuracy	98.71 ± 0.48	97.89 ± 0.12	93.68 ± 5.28	93.74 ± 0.73	99.42 ± 0.26	91.29 ± 1.08	98.60 ± 1.25
Precision 0	99.41 ± 0.38	99.64 ± 0.30	89.82 ± 8.58	100.00 ± 0.00	99.53 ± 0.44	100.00 ± 0.00	98.55 ± 2.36
Precision 1	98.05 ± 0.85	96.27 ± 0.26	99.59 ± 0.58	88.89 ± 1.16	99.30 ± 0.23	85.19 ± 1.54	98.73 ± 1.10
Average Precision	98.73 ± 0.47	97.96 ± 0.12	94.71 ± 4.36	94.45 ± 0.58	99.42 ± 0.26	92.59 ± 0.77	98.64 ± 1.19
Recall 0	98.01 ± 0.88	96.14 ± 0.29	99.65 ± 0.47	87.49 ± 1.46	99.30 ± 0.23	82.57 ± 2.17	98.71 ± 1.13
Recall 1	99.42 ± 0.37	99.65 ± 0.29	87.72 ± 10.47	100.00 ± 0.00	99.53 ± 0.44	100.00 ± 0.00	98.48 ± 2.50
Average Recall	98.71 ± 0.48	97.89 ± 0.12	93.68 ± 5.28	93.74 ± 0.73	99.42 ± 0.26	91.29 ± 1.08	98.60 ± 1.25
F-measure 0	98.70 ± 0.48	97.86 ± 0.12	94.27 ± 4.75	93.32 ± 0.83	99.41 ± 0.26	90.44 ± 1.32	98.61 ± 1.21
F-measure 1	98.72 ± 0.47	97.93 ± 0.12	92.96 ± 5.95	94.12 ± 0.65	99.42 ± 0.26	91.99 ± 0.91	98.58 ± 1.28
Average F-measure	98.71 ± 0.48	97.89 ± 0.12	93.61 ± 5.35	93.72 ± 0.74	99.42 ± 0.26	91.22 ± 1.11	98.60 ± 1.25

Metrics for the negative and positive classes are suffixed by 0 and 1, respectively, e.g., Precision 0, Recall 1, etc.

**Figure 3:**
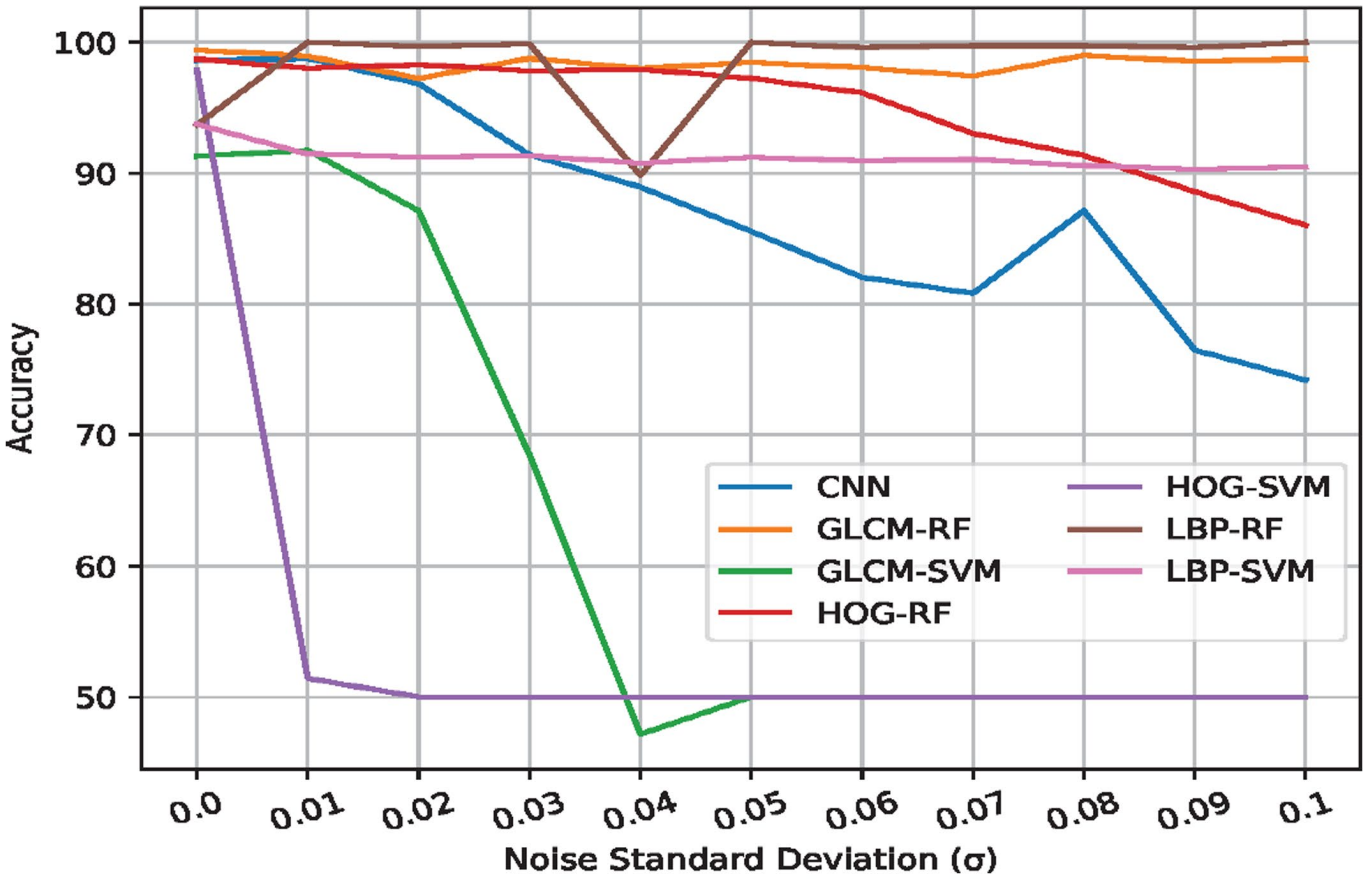
Effect of noise on predictive performance - accuracy.

**Figure 4:**
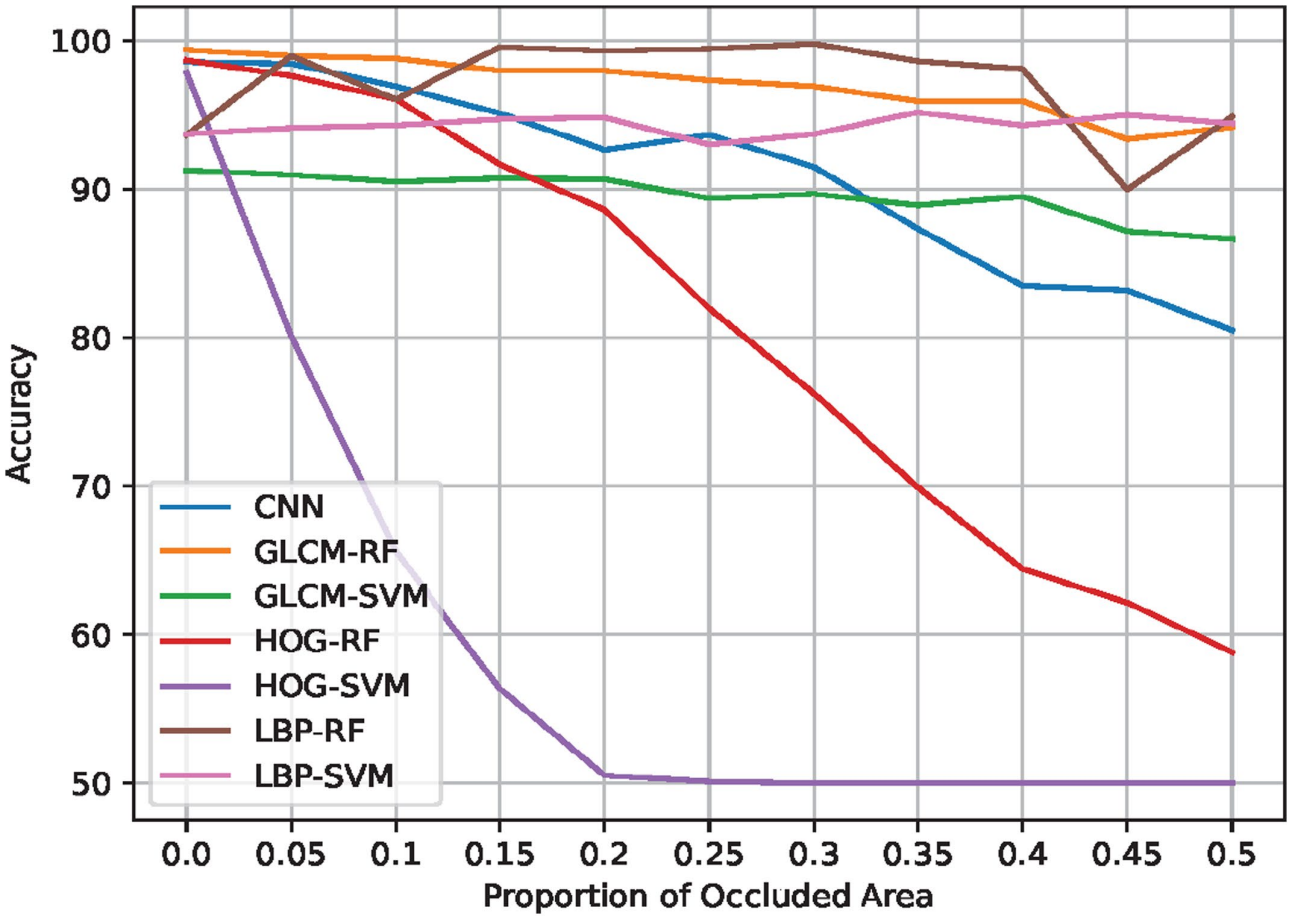
Effect of occlusion on predictive performance-accuracy.

Occlusion and noise robustness tests were conducted to assess the resilience of each model. As shown in Tables 4, 5, these tests simulate real-world conditions where images might be partially obscured or contain noise. Figures 3, 4 illustrate the effects of occlusion and added Gaussian noise, respectively, on the performance of seven possible combinations of feature extraction and ML methods. The CNN, notably, does not require feature extraction for these tests.

**Table 4:** Results for noise robustness experiment.

Metric	Method						
	HOG+RF	HOG+SVM	LBP+RF	LBP+SVM	GLCM+RF	GLCM+SVM	CNN
Accuracy	86.02 ± 3.83	50.00 ± 0.00	100.0 ± 0.00	90.47 ± 1.12	98.71 ± 1.09	50.00 ± 0.00	74.21 ± 15.57
Precision 0	79.28 ± 5.42	50.00 ± 0.00	100.0 ± 0.00	100.00 ± 0.00	100.00 ± 0.00	50.00 ± 0.00	90.00 ± 20.00
Precision 1	97.92 ± 1.24	0.00 ± 0.00	100.0 ± 0.00	84.02 ± 1.59	97.54 ± 2.08	0.00 ± 0.00	58.69 ± 30.85
Average Precision	88.60 ± 2.45	25.00 ± 0.00	100.0 ± 0.00	92.01 ± 0.80	98.77 ± 1.04	25.00 ± 0.00	74.34 ± 25.13
Recall 0	98.36 ± 1.01	100.00 ± 0.00	100.0 ± 0.00	80.94 ± 2.24	97.43 ± 2.18	100.0 ± 0.00	68.42 ± 25.15
Recall 1	73.68 ± 8.33	0.00 ± 0.00	100.0 ± 0.00	100.00 ± 0.00	100.00 ± 0.00	0.00 ± 0.00	80.00 ± 40.00
Average Recall	86.02 ± 3.83	50.00 ± 0.00	100.0 ± 0.00	90.47 ± 1.12	98.71 ± 1.09	50.00 ± 0.00	74.21 ± 15.57
F-measure 0	87.68 ± 3.00	66.67 ± 0.00	100.0 ± 0.00	89.45 ± 1.36	98.68 ± 1.11	66.67 ± 0.00	71.56 ± 17.45
F-measure 1	83.80 ± 5.06	0.00 ± 0.00	100.0 ± 0.00	91.31 ± 0.94	98.74 ± 1.06	0.00 ± 0.00	67.35 ± 34.30
Average F-measure	85.74 ± 4.03	33.33 ± 0.00	100.0 ± 0.00	90.38 ± 1.15	98.71 ± 1.09	33.33 ± 0.00	69.46 ± 21.62

**Table 5:** Results for occlusion robustness experiment (occluded area proportion = 0.5).

Metric	Method						
	HOG+RF	HOG+SVM	LBP+RF	LBP+SVM	GLCM+RF	GLCM+SVM	CNN
Accuracy	58.83 ± 1.52	50.00 ± 0.00	94.91 ± 9.18	94.44 ± 1.06	94.21 ± 1.33	86.67 ± 1.15	80.53 ± 4.61
Precision 0	54.87 ± 0.92	50.00 ± 0.00	92.95 ± 12.48	100.00 ± 0.00	90.24 ± 1.87	100.00 ± 0.00	83.37 ± 5.74
Precision 1	98.92 ± 2.16	0.00 ± 0.00	99.88 ± 0.23	90.03 ± 1.68	99.08 ± 0.53	78.97 ± 1.44	78.76 ± 5.85
Average Precision	76.90 ± 1.04	25.00 ± 0.00	96.42 ± 6.21	95.02 ± 0.84	94.66 ± 1.18	89.49 ± 0.72	81.06 ± 4.66
Recall 0	99.77 ± 0.47	100.00 ± 0.00	99.88 ± 0.23	88.89 ± 2.12	99.18 ± 0.47	73.33 ± 2.30	76.61 ± 8.31
Recall 1	17.89 ± 3.19	0.00 ± 0.00	89.94 ± 18.41	100.00 ± 0.00	89.24 ± 2.24	100.00 ± 0.00	84.44 ± 5.53
Average Recall	58.83 ± 1.52	50.00 ± 0.00	94.91 ± 9.18	94.44 ± 1.06	94.21 ± 1.33	86.67 ± 1.15	80.53 ± 4.61
F-measure 0	70.80 ± 0.74	66.67 ± 0.00	95.81 ± 7.41	94.10 ± 1.21	94.50 ± 1.22	84.60 ± 1.52	79.57 ± 5.52
F-measure 1	30.17 ± 4.60	0.00 ± 0.00	93.48 ± 12.02	94.75 ± 0.94	93.89 ± 1.45	88.24 ± 0.90	81.30 ± 3.98
Average F-measure	50.48 ± 2.66	33.33 ± 0.00	94.64 ± 9.72	94.43 ± 1.07	94.19 ± 1.33	86.42 ± 1.21	80.43 ± 4.67

We present our results in Table 3. For the sake of the clarity of the analysis, we limit the discussion to aggregate insights centered around the accuracy per method. In general, the different approaches considered show high predictive performance across the board. This can confirm their general efficacy as mentioned previously. Furthermore, the results also generally show very small amounts of variance, indicating their stability. This suggests that the results are reliable and statistically consistent overall.

SVM-based methods generally show lower performance relative to their RF-based counterparts. On the other hand, the RF-based methods and deep CNN classifier provide competitive performance. This can be attributed to the ensemble nature of the former, and the flexibility of the latter which permits it to model complex, nonlinear relationships with high fidelity. However, all three methods provide near-similar performance. Notably, however, the GLCM+RF, HOG+RF, and CNN methods show the highest predictive performance. This superiority is also reflected in the secondary performance metrics, where they show balanced/consistent performance across both classes. This contrasts with other methods, e.g., LBP-RF, LBP-SVM, and GLCM-SVM, which show large disparities in per-class performance.

To further validate our findings, we present and discuss additional experiments that assess the system’s performance under challenging conditions, including the impact of noise and occlusion on prediction accuracy.

### 4.1 Noise robustness

We begin by considering the effect of noise on our system’s predictive performance. That is, after normal training, we investigate how noise contamination during inference (which can occur during real-world image acquisition) affects the recognition ability of our proposed system.

**Figure 5:**
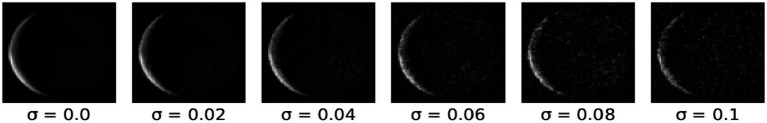
Effect of considered noise levels on input images.

To evaluate this, we use Gaussian noise (with zero mean) at some standard deviation (*σ*) to contaminate the images used for testing. For full coverage, we consider a range of σ values ranging from 0 (no noise) to 0.1. A visualization showing the impact of the considered noise levels is provided in Figure 5.

Similar to our main experiments, we consider the same set of predictive models. To streamline the subsequent discourse, we focus on accuracy as the performance metric of interest, and present the results graphically in Figure 3, where each line represents one type of predictive model. We also provide quantitative results at the experiment extreme (i.e., *σ* = 0.1) in Table 4, which shows the secondary metrics in the extreme case.

From Figure 3, it can be seen that the various predictive models considered are generally robust to noise, except for the HOG-SVM combination, which degrades to random guessing at very small noise levels (∼ 0.01). This can be attributed to the fact that noise changes the texture of images, which leads to changes in the values of texture feature descriptors for such images (as considered in this work). Given that the SVM classifier relies on higher dimensional transformation, these changes are amplified in the higher-dimensional space, severely affecting performance. Notably, the same HOG descriptor – when used with a RF-based classifier - does not show the same level of degradation. This is because the RF-based classifier utilizes bootstrap aggregation (Loucif et al., 2024) which involves sampling random features per constituent decision tree, and multiple decision trees. In practice, this has a smoothing effect, i.e., since it involves random feature subsets, there is less overall disruption in the subsets than in the overall feature space, granting superior noise robustness. Similar to the initial results, RF-based models and CNN show the best performance and superior stability in general.

### 4.2 Occlusion robustness

**Figure 6:**
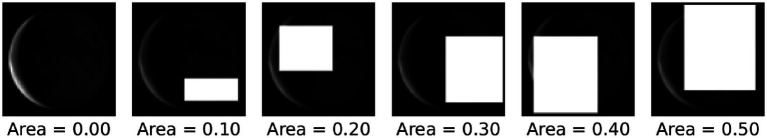
Sample images occluded via random erasure. For illustration, white pixels demarcate the occluded regions.

We also consider the effect of occlusions on our proposed system, since in practice the moon could be occluded by clouds or other aerial obstacles during the image acquisition process. To simulate this, we consider Random Erasure (Zhong et al., 2020), which ‘blacks out’ (i.e., sets pixel values to 0) a rectangular region of a specified proportion in the input image. The location and aspect ratio of the blacked-out region are selected randomly. We provide a visual example of Random Erasure’s operation in Figure 6. Note that we use white pixels to demarcate the occluded regions for illustration purposes. In practice, the occluded regions are filled with black pixels.

Therefore, we experiment with increasing the area of the occluded region from 0 (no occlusion) to 0.5 (half of the image area is occluded). As with preceding experiments, we repeat experiments at each occlusion area value 5 times and report the mean performance for each method. We also consider accuracy as the main performance metric, and present the results graphically, shown in Figure 6, and quantitatively in the extreme case, Table 5, as in the preceding section.

From the figure, increasing the proportion of the occluded area generally degrades performance across all the considered methods as expected. Similar to the preceding experiment, HOG-SVM shows the fastest rate of degradation, dropping to 50% (i.e., same as random guessing) at 20% occlusion area.

On the other hand, LBP-RF and GLCM-RF show the most robustness, retaining more than 90% of the performance even at the maximum occlusion area. These findings are consistent with those found when no noise and no occlusion considered, indicating the superior efficacy of these two models relative to the others considered in this study. This motivates the use of these models in practical settings, especially considering their simplicity.

Despite the promising results, we acknowledge a limitation of this study related to the use of space-borne imagery and astronomical parameters, which do not account for certain conditions such as atmospheric effects, regional localization, and human observer variability that may influence real-world crescent sighting from Earth. Regarding societal implications, the identification of the new crescent holds significant religious and cultural importance, especially for religious events. The proposed approach is not intended to replace traditional techniques such as naked-eye sighting, but rather to serve as a supportive, data-driven tool for observation and decision-making.

## 5 Conclusion and future work

This paper presented new approach to enhance the identification of the new crescent employing both ML and DL techniques. We used the LRO data extracted from NASA while applying different techniques such as CNN, RF, and SVM. Those models have reflected significant improvements in identifying patterns related to visibility of the new crescent moon. We found that CNN along with the RF techniques had shown the best accurate prediction results compared to the other techniques, they have demonstrated promising results even when noise and occlusion were introduced into the images.

The proposed approach is not intended to replace the current traditional moon sighting techniques, but it can be used as a supportive method to complement those currently existing traditional methods. They offer an additional support to standardize the process and assist in resolving some discrepancies in the lunar calendar calculations across different regions in the world. While our results have demonstrated effective detection of the birth of new crescent moon in LRO images, real-world hilāl sighting from earth also depends on many aspects such as the atmosphere, optical, and observer-related factors that are not modeled in this study. This work provides a foundational step toward automated lunar phase analysis and may support future efforts that integrate terrestrial observational constraints.

For future work, more datasets can be expanded to include images related to Earth-based observational imagery, which will allow the framework to be extended to different geographical locations and observational conditions that can enhance the applicability of the proposed model. The latter can be further refined to incorporate more diverse environmental factors, such as light pollution data and different weather patterns. Additionally, as this is the first exploratory study to apply ML and DL techniques for identifying the birth of the new crescent moon, this work lays the groundwork for applying more advanced DL techniques, other than CNN, to further enhance the identification of the new crescent, especially under challenging observational conditions. Moreover, future studies will explicitly incorporate explainability methods to validate that model attention is focused on the crescent region and to address broader ethical, transparency, and trust-related considerations, once the core detectability framework is fully established.

## Data Availability

Publicly available datasets were analyzed in this study. The datasets used and/or analyzed during the current study are available from the corresponding author on reasonable request.
